# Data from human salivary proteome – A resource of potential biomarkers for oral cancer

**DOI:** 10.1016/j.dib.2015.06.014

**Published:** 2015-07-02

**Authors:** Priya Sivadasan, Manoj Kumar Gupta, Gajanan J. Sathe, Lavanya Balakrishnan, Priyanka Palit, Harsha Gowda, Amritha Suresh, Moni Abraham Kuriakose, Ravi Sirdeshmukh

**Affiliations:** a Head and Neck Oncology, Mazumdar Shaw Medical Center, Narayana Health, Bangalore 560099, India; b Institute of Bioinformatics, International Tech Park, Bangalore 560066, India; c Mazumdar Shaw Center for Translational Research, Mazumdar Shaw Medical Foundation, Narayana Health, Bangalore 560099, India

## Abstract

Salivary proteins are an important source for developing marker-based assays for oral cancers. To get an insight into the proteins present in human saliva, we applied multiple strategies involving affinity-based depletion of abundant proteins, fractionation of the resulting proteins or their tryptic peptides followed by LC–MS/MS analysis, using high resolution mass spectrometry. By integrating the protein identifications observed by us with those from similar workflows employed in earlier investigations, we compiled an updated salivary proteome. We have mapped the salivary proteome to the published data on differentially expressed proteins from oral cancer tissues and also for their secretory features using prediction tools, SignalP 4.1, TMHMM 2c and Exocarta. Proteotypic peptides for the subset of proteins implicated in oral cancer and mapped to any two of the prediction tools for secretory potential have been listed. The data here are related to the research article “Human saliva proteome – a resource of potential biomarkers for oral cancer” in the Journal of Proteomics [1].

## 1 Value of the data

• Proteins identified, compiled from published LC–MS/MS analysis and the data from our recent analysis represent an updated salivary proteome.
• The list of salivary sub-proteome includes proteins which are reported to be differentially expressed in oral cancer tissue specimens and have secretory potential.
• A high confidence list of proteins along with their proteotypic peptides is supported by their relevance in oral cancer and predicted secretory features.
• This subset would serve as an important reference for developing targeted analysis for clinical applications.

**Table:** 

Specifications table	
Subject area	Biology

More specific subject area	Saliva proteomics or proteome
Type of data	Tables, excel files
How data was acquired	Fourier Transform LTQ-Orbitrap Velos mass spectrometer (Thermo Fischer Scientific, Bremen, Germany) equipped with Proxeon Easy nLC was used for LC–MS/MS analysis
	Proteome Discoverer 1.4 and SEQUEST search engine Human RefSeq 60 database
	Human Oral Microbiome Database (HOMD)
Data format	Analyzed
Experimental factors	Human saliva proteomic analysis, processing and fractionation of salivary proteins, mass spectrometry, data analysis
Experimental features	Human saliva from healthy subjects was subjected to depletion of high abundant proteins by starch affinity and/or antibody affinity for plasmatic proteins or enrichment of low abundant proteins by capturing with hexapeptide library. Pre-fractionation of proteins by SDS-PAGE followed by in-gel tryptic digestion or SCX chromatography of tryptic peptides from in-solution digested total proteins. Mass spectrometry was carried out using high resolution MS platform.
Data source location	Bangalore, India
Data accessibility	Analyzed datasets are directly provided with this article

## 2 Data, experimental design, materials and methods

### 2.1 Sample collection and processing

The study was approved by the Institutional Ethics Committee. The procedure for collection and processing of saliva was adapted from earlier reports [2,3]. Briefly, unstimulated saliva samples (5 ml) were collected from healthy subjects of either sex in the age group between 20 and 50 years, with written informed consent. The individuals selected were without any risk habits like tobacco chewing, smoking or alcohol abuse. Samples were collected in the morning after rinsing the mouth with water and with subjects refraining from food/drink for at least 1 h prior to the collection. All the samples were centrifuged at 2000 rpm, at 4 °C for 10 min to remove the cells. The supernatant was then collected and centrifuged at 14,000 rpm to remove any debris. Protein estimation was carried out using RC-DC protein assay (Bio-Rad, USA) as per the manufacturer׳s guidelines and the samples were stored at −80 °C until further use.

### 2.2 Depletion and fractionation methods

Equal volumes of saliva were pooled based on the age groups and pooled saliva samples were processed further. One pool included samples from individuals of 30–50 years of age, (Pool A) and the other pool included samples from individuals of 20–30 years of age (Pool B). We adopted two strategies to deplete abundant proteins. Depletion of amylase alone was carried out by using starch affinity-based amylase capture and depletion of amylase and plasmatic proteins by amylase capture followed by antibody-based depletion of plasma proteins such as albumin, immunoglobulins and others. The depleted protein fraction was then subjected to fractionation on SDS-PAGE and in-gel tryptic digestion or in solution digested with trypsin and tryptic peptides were fractionated by SCX chromatography. In another strategy, compression of the protein dynamic range of total salivary proteins was carried out using hexapeptide library enrichment kit (ProteoMiner, Bio-Rad, CA, USA). The tryptic digest of the enriched protein fraction was then subjected to fractionation by SCX chromatography (See figure 1 in Ref [1] for details).

For amylase depletion, 5 ml of pooled saliva (approximately 5 mg of protein) was mixed with 1.5 g of potato starch (Sigma Aldrich, MO, USA) [previously washed 3 times with water (3000 rpm, 5 min)] and incubated for one hour in a rotating shaker, at room temperature. The mixture was then centrifuged at 3000 rpm for 5 min and the supernatant was collected. The pellet was washed again to recover trapped saliva. Protein estimation was then carried out as mentioned above. Depletion of albumin, immunoglobulins and any other abundant plasma proteins (transferrin, fibrinogen, immunoglobulin A, haptoglobin, alpha antitrypsin, alpha 2 macroglobulin, immunoglobulin M, apolipoprotein A1, alpha1 acid glycoprotein, Complement C3, apolipoprotein A11 and transthyretin) was carried out using Human MARS-14 spin cartridge (Agilent Technologies, CA, USA ) as per manufacturers׳ instructions. The protein sample after amylase depletion was passed through the MARS-14 cartridge and the unbound protein was collected. The procedure was repeated multiple times to collect approximately 500 μg of depleted protein fraction for further experiments. Flow through fractions were collected, concentrated and desalted using a 5 kDa MW cut off ultracentrifugal filter device (Amicon, Millipore, Billerica, MA). The protein concentration of the sample was determined as mentioned above.

Two hundred micrograms of the above mentioned depleted saliva protein was resolved on a 10% SDS-PAGE (16×18 cm^2^) and the gel was stained using colloidal Coomassie blue. Twenty five gel slices were excised and destained using 40 mM ammonium bicarbonate in 40% acetonitrile (ACN). The sample was then subjected to reduction using 5 mM DTT (60 °C for 45 min) followed by alkylation using 20 mM iodoacetamide (10 min. at room temperature). In-gel digestion with trypsin was carried out at 37 °C for 12–16 h using modified sequencing grade Trypsin (Promega, WI, US). Peptides were extracted from gel pieces sequentially using 5% formic acid, 5% formic acid in 40% ACN and finally with 100% ACN. The extracted peptides were dried and stored at −80 °C until LC–MS/MS analysis.

Alternatively, depleted protein fraction was subjected to direct in-solution digestion with trypsin and the resulting peptides were fractionated by SCX chromatography. Briefly, 200 µg of protein was reduced with 5 mM DTT and alkylated using 10 mM IAA as above. The proteins were then digested with trypsin as above and the digested peptide mix was reconstituted in solvent A (10 mM potassium phosphate, 30% ACN, pH 2.7) and fractionation was carried out on a SCX column (Polysulfoethyl A column; 300 Å, 5 µm, 100×2.1 mm^2^; PolyLC, MD, USA) using 1200 HPLC system (Agilent Technologies, CA, USA) coupled with a binary pump, UV detector and a fraction collector. Peptides were eluted using a linear salt gradient (0 to 35%) of solvent B (10 mM potassium phosphate buffer containing 30% ACN, 350 mM KCl, pH 2.7) at a flow rate of 200 µl/ min. The adjacent fractions were then pooled based on the chromatographic profile to make the total number to 25. The samples were dried, reconstituted in 0.1% TFA and desalted using C18 stage-tip. The desalted samples were dried and stored at −80 °C until further analysis.

For enrichment using ProteoMiner, salivary proteins were subjected to the procedure according to the manufacturers׳ instructions (ProteoMiner; Bio-Rad, CA, USA). Briefly, 10 mg of salivary protein was added to the ProteoMiner column, incubated in a rotational shaker for 2 h at room temperature and centrifuged at 1000*g* for 1 min to discard the unbound fraction. The column was then washed thrice with 200 µl of wash buffer, by centrifugation at 1000 g for 1 min. Two hundred microlitres of deionized water was added and centrifuged at 1000 g for 1 min. The enriched low abundant proteins bound to the column were eluted with 100 µl of rehydrated elution reagent, desalted using 5 kDa MW cut off ultracentrifugal filter device (Amicon, Millipore, Billerica, MA) and protein estimation was carried out. The enriched protein sample was digested in-solution with trypsin and the tryptic digest was subjected to SCX fractionation as described above.

### 2.3 LC–MS/MS analysis

Fourier-Transform LTQ-Orbitrap Velos mass spectrometer (Thermo Fischer Scientific, Bremen, Germany) equipped with Proxeon Easy nLC was used for LC–MS/MS analysis. In house chromatographic capillary columns made up of Magic C_18_ AQ reversed phase material (Michrom Bioresources, 5 and 3 μm, 100 Å) were used for HPLC. Nanospray source with an emitter tip of 10 µm (New Objective, Woburn, MA) was used for ionization with a voltage of 2 kV. Peptides were enriched on trap column (75 mm×2 cm) at a flow rate of 3 µL/min using Solvent A (0.1% formic acid) followed by fractionation in an analytical column (75 mm×10 cm) to resolve the peptides. A linear gradient of 7–30% solvent B (0.1% formic acid, 95% ACN) was used at a flow rate of 350 nL/min., for 80 min. The mass spectrometry parameters used are as follows: acquisition of the full scan data was implemented with a mass resolution of 60,000 at 400 m/z, top 20 intense peaks from each MS cycle were selected for MS/MS fragmentation with a mass resolution of 15,000 at 400 m/z. Only multiple charged peptides were selected and 39% normalized collision energy was used for fragmentation with 45 s exclusion time. Automatic gain control and filling time were kept at 5×10^5^ ions and 100 ms for MS, and 1×10^5^ ions and 500 ms for MS/MS, respectively. Polydimethylcyclosiloxane (m/z, 445.1200025) ion was used for internal calibration [4].

### 2.4 Protein identification and bioinformatics analysis

Mass spectrometry data was analyzed using Proteome Discoverer v1.4software (Thermo Scientific, Bremen, Germany). Peak list file generation and database searches were carried out in SEQUEST mode. Precursor mass range of 350–8000 Da and signal to noise ratio of 1.5 were used as the criteria for generation of peak list files. Database searches for protein identifications were carried out for human proteins using, NCBI Human RefSeq 60 protein database. As human saliva also contains microbial flora, a separate search was also carried out using combined database of NCBI Human RefSeq60 and oral microbial proteins from the Human Oral Microbiome Database (HOMD; www.homd.org). We used the searches against human protein database alone to identify all human proteins. The identifications were compared with those from the combined database search and any shared peptides of microbial protein origin identified were filtered out to ensure that human protein identifications were completely based on unique human peptides and microbial protein identifications were based on unique microbial peptides. The human protein identifications from each of the 4 workflows used are provided in Tables 1A–D. The list of non-redundant human and microbial proteins identified from all the 4 workflows is provided in Tables 2 and 3, respectively.

The parameters used for database searches included trypsin as a protease with one missed cleavage, carbamidomethyl cysteine as a fixed modification, and oxidation of methionine as a dynamic modification. Precursor ion and fragment ion mass error window used was 20 ppm and 0.1 Da, respectively. The proteins and their corresponding peptide list were obtained using the criteria: peptide confidence – high; peptide rank – 1; Xcorr filters at individual MS runs to allow 1% FDR at peptide level with searches using decoy database. Only unique peptides were considered for protein identifications. Further, all the single peptide identifications were manually screened for the quality of spectra, peptide length and uniqueness. The single peptide/protein hits were included only if the fragmentation was scored as good with respect to 70–80% of ‘b’ ion or ‘y’ ion information with optimal intensities and the peptides were at least 6 residues long. Peptides, which have ambiguous spectra, were not included for valid identifications.

Gene Ontology (GO) classification was done using HPRD (http://www.hprd.org) to classify identified proteins for their subcellular localization, molecular function and biological processes (See figure 2 in Ref. [1]). SignalP 4.1 (www.cbs.dtu.dk/services/SignalP) and TMHMM 2.0c (http://www.cbs.dtu.dk/ services/TMHMM) were used to predict signal peptide or transmembrane domain presence in the proteins identified. The proteins were also compared with human exosomal protein database (Exocarta; http://exocarta.org) [5].

#### 2.4.1 Compilation of salivary proteome

Mass spectrometry-based proteomic studies using whole saliva or glandular saliva, varied depletion and fractionation methods and instrumentation platforms (LC-MALDI TOF/TOF, LTQ-linear ion trap, LTQ-Orbitrap XL and QSTAR Pulsar XL instruments) which varied in their analytical capabilities, were reported by several research groups. [6–10]. Comprehensive cataloging of the salivary proteome was done by combining the data from these earlier LC–MS/MS based studies on saliva along with the data from our study (Table 4; also see figure 3 in Ref. [1]). Gene Ontology classification and their secretory potential analysis was carried out using the bioinformatics tools described above. Comparing the updated human salivary proteome compiled and the differentially expressed proteins from oral cancer tissues from published literature, we identified proteins implicated in oral cancer **(**Table 5**)**. Further, the secretory potential of these proteins was assessed based on the three criteria as described above i.e, exosomal, signal peptide and transmembrane domain. This combined list of oral cancer relevant proteins which also map to secretory potential is given in Table 5. From these, high confidence secretory proteins were sorted that matched to atleast two of the three secretory parameters. The proteotypic peptides/most observed peptides of these proteins were selected from the Global Proteome Machine Database (GPMdb), along with their additional peptides consistently observed in the multiple analysis datasets in the salivary proteome. They are provided in Table 6 as high confidence list for targeted analysis.
